# Effect of Mid-infrared Free-Electron Laser Irradiation on Refolding of Amyloid-Like Fibrils of Lysozyme into Native Form

**DOI:** 10.1007/s10930-012-9452-3

**Published:** 2012-10-11

**Authors:** Takayasu Kawasaki, Jun Fujioka, Takayuki Imai, Koichi Tsukiyama

**Affiliations:** IR Free Electron Laser Research Center, Research Institute for Science and Technology (RIST), Tokyo University of Science, 2641, Yamazaki, Noda, Chiba 278-8510 Japan

**Keywords:** Aggregation, Amide bands, Amyloid fibril, Free electron laser, Refolding

## Abstract

Aggregation of lysozyme in an acidic solution generates inactive amyloid-like fibrils, with a broad infrared peak appearing at 1,610–1,630 cm^−1^, characteristic of a β-sheet rich structure. We report here that spontaneous refolding of these fibrils in water could be promoted by mid-infrared free-electron laser (mid-IR FEL) irradiation targeting the amide bands. The Fourier transform infrared spectrum of the fibrils reflected a β-sheet content that was as low as that of the native structure, following FEL irradiation at 1,620 cm^−1^ (amide I band); both transmission-electron microscopy imaging and Congo Red assay results also demonstrated a reduced fibril structure, and the enzymatic activity of lysozyme fibrils recovered to 70–90 % of the native form. Both irradiations at 1,535 cm^−1^(amide II band) and 1,240 cm^−1^ (amide III band) were also more effective for the refolding of the fibrils than mere heating in the absence of FEL. On the contrary, either irradiation at 1,100 or 2,000 cm^−1^ afforded only about 60 % recovery of lysozyme activity. These results indicate that the specific FEL irradiation tuned to amide bands is efficient in refolding of lysozyme fibrils into native form.

## Introduction

In many instances, protein aggregation processes accompanied by decrease of solubility lead to suppression of protein functions such as enzymatic activities, a putative cause of several diseases in humans. For example, amyloid fibrils are formed by amyloid beta Aβ (40–43) [7, 21], tau protein [11], polyglutamine [22], transthyretin [35], α-synuclein [24], prion protein [19], and β_2_-microgulobulin [15]. In addition, it has been recently reported that sequentially non-homologous proteins, insulin [34], proinsulin c-peptide [14], lysozyme [6], calcitonin [8], and myoglobin [3] may form amyloid-like fibrils under certain conditions. These observations imply that amyloid structures are common to the intrinsic structures of various proteins, although the mechanism of their formation is not completely understood. According to recent studies investigating short peptides (which have a tendency to form amyloid fibrils), the aggregation reaction is purportedly caused by ionic amino acid residues such as lysine and glutamic acid, while hydrophobic interactions between aromatic amino acids have been proposed to be the promotion factors for fibril formation via π–π interactions [20, 26]. The ionic interactions between acidic and basic (i.e., arginine) residues have also been proposed to play an important role in the formation of toxic soluble oligomers of Aβ42 [10, 33]. An inhibition experiment using β-sheet breaker peptides indicates that the aggregation process of amyloid is reversible [23]; therefore, it is hypothesized that the fibrils are spontaneously refolded into their native form by dissociation of the aforementioned non-covalent bonds of the β-sheet clusters, or incorporation of water molecules into the hydrophobic core of the fibrils [25].

A mid-infrared free-electron laser (mid-IR FEL) can excite the specific bonds within mid-IR region, including amide bands, accounting for its use in multi-photon dissociation reactions [4, 27] and ablation of biological tissues and various proteins [1, 5, 28, 30, 31]. Investigators in Vanderbilt University have performed mid-IR FEL-induced ablation of cornea tissue, observing secondary structural changes and peptide fragmentation of collagen [29, 36]. In addition, Oomens et al. [17] observed the detachment of potassium ions from proteins in the gas phase following the irradiation with FEL at 6 μm (1,667 cm^−1^) by using Fourier transform ion cyclotron resonance mass spectrometry. From these studies, it may be concluded that FEL irradiation can induce major changes in the higher-order structure of proteins. In the present study, we targeted the amyloid fibrils for mid-IR FEL irradiation. During the amyloid-formation process, vibrational modes of amide bonds, including C=O stretch, C–N stretch, and N–H bend, are affected by β-sheet formation and are observed by FTIR spectra [2, 9]. Variables that influence the infrared data include hydrogen bonding, ionic interactions, and hydrophobic interactions formed during the course of β-sheet cluster formation. It has been hypothesized that these non-covalent interactions in the fibrils can be affected by FEL irradiation. In this study, we selected lysozyme as a model protein for mid-IR FEL irradiation, because the enzyme is commercially available in abundance and has been reported to form amyloid-like fibrils under specific acidic conditions in vitro [13]. Here, we report that dissociation of amyloid-like fibrils of lysozyme into the native form of the protein could be promoted by mid-IR FEL irradiation at the amide band.

## Materials and Methods

### Materials

Chicken egg white lysozyme, *Micrococcus lysodeikticus* ATCC 4698, phosphotungstic acid, and Congo Red were purchased from Sigma-Aldrich (Tokyo, Japan). Acetic acid and sodium chloride (NaCl) were purchased from Wako Pure Chemical Industries (Osaka, Japan). The KBr-mini-plate was purchased from Jasco Engineering Co. (Tokyo, Japan).

### Preparation of Lysozyme Fibrils

Lysozyme powder was dissolved to a concentration of 2.5 mg/mL in H_2_O (1 mL) containing 20 % acetic acid and 0.5 M NaCl, and incubated for 20 h at 37 °C. The resulting aggregates were precipitated by centrifugation at 14,000 rpm for 10 min at room temperature, washed by the addition of 0.5 mL of distilled water, and dried. The fibrils generated were re-suspended in distilled water and were analyzed by a variety of methods described in the subsequent sections.

### Method of Mid-infrared Free-Electron Laser Irradiation at the Tokyo University of Science (FEL-TUS)

**Fig. 1:**
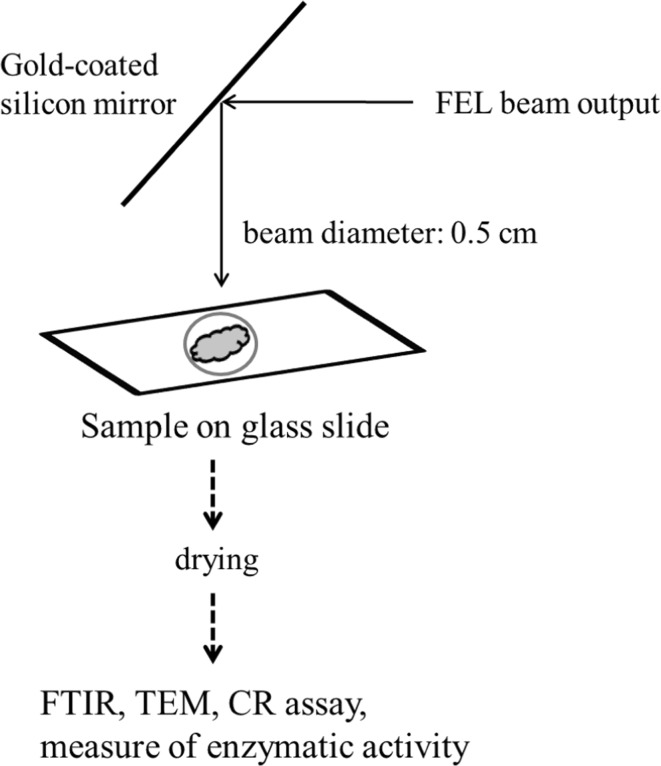
Outline of the present methods. The FEL beam output is transported through the vacuum tube and controlled by the mirror to be directed onto the sample. The spot size of beam line is ca. 0.5 cm in diameter. The lysozyme sample was placed within the *circle* of beam spot on the slide glass and irradiated with the FEL. The sample was air-dried and subsequently analyzed by the various methods as described in the experimental section

Overall process in the present study is shown in Fig. 1. The structure and the features of FEL-TUS have been described in previous studies [16, 27]. In brief, FEL is an apparatus that generates a laser beam using synchrotron radiation as a seed, with a variable wavelength within the mid-infrared region of 5–16 μm (625–2,000 cm^−1^). FEL-TUS provides 2 types of laser pulses, macro-pulse and micro-pulse. The macro-pulse has duration of ~2 μs and a repetition rate of 5 Hz throughout the operation, consisting of a train of micro-pulses with duration of 2 ps. The interval of 2 consecutive micro-pulses is 350 ps, which corresponds to the RF frequency (2,856 MHz) employed for the linear accelerator. Accordingly, one macro-pulse contains ~6,000 micro-pulses. The energy of the laser pulse used for the current experiment was in the range of 6–10 mJ macropulse^−1^. The lysozyme fibrils in a water suspension on a glass slide at 37 °C were irradiated with the output of the FEL tuned to various wavelengths. To avoid the vaporization of water, fresh 10 μL water was periodically added to the suspension during the irradiations. After the irradiation was completed, the sample on the glass was dried and subjected to the various analyses.

### Fourier-Transform Infrared Spectroscopy

Fourier transform infrared spectra were recorded on an FT/IR 615 spectrophotometer (Jasco International Co., Ltd., Tokyo, Japan) by using a solid KBr mini-plate. The protein sample was mixed with KBr pellet and a thin plate was prepared, and the measurements were performed using 16 scans under 4 cm^−1^ resolutions. Secondary structures of the lysozyme samples were estimated by using the bundled protein analysis software (IR-SSE; JASCO Co., Ltd.), which was developed for evaluation of protein conformational changes in biological tissue [32].

### Sodium Dodecyl Sulfate Polyacrylamide Gel Electrophoresis (SDS-PAGE)

The lysozyme samples (approximately 1.0 mg/mL) were mixed with sodium dodecyl sulfate (SDS) sample buffer (25 mM of Tris, 192 mM of glycine, and 0.1 % of SDS) and loaded onto a 15 % polyacrylamide gel (ATTO Corporation, Tokyo, Japan). The migrated proteins on the gel were detected by performing Coomassie staining.

### Transmission Electron Microscopy

Specimens for transmission electron microscopic (TEM) observation were prepared as follows: First 2 μL of each lysozyme sample was deposited onto copper grids (200 mesh; Nisshin EM Co., Ltd, Tokyo, Japan) covered with collodion film hydrophilized by an electric glow discharge. After 30 s of deposition, any excess amount of the sample was blotted out using a filter paper, followed by 2 deposition-blotting cycles with 20 μL of water and 2 additional cycles of phosphotungstic acid (25 μL of 1 % w/v). Prior to sample preparation, the staining solution was filtered using a 0.22-μm membrane filter to remove large crystals. The TEM observation was performed using a Hitachi H-7650 (Tokyo, Japan) at 120 kV of accelerating voltage.

### Congo Red Binding Assay

The absorbance peak of Congo Red is known to shift from 490 to 510 nm in the presence of the fibrils [12]. Aliquots of lysozyme solution (30 μL) were added to an equivalent volume of Congo Red solution (0.2 mM in PBS) and incubated for 10 min at room temperature. The resulting absorbance values were obtained from a 400 to 700 nm scan using a multi-label counter (PerkinElmer, Tokyo, Japan).

### Measurements of Lysozyme Activities

Lysozyme activity was defined as follows, based on the product information from Sigma-Aldrich. In brief, the substrate, a cell wall extract of *Micrococcus lysodeikticus* (Sigma), was dissolved in PBS (100 μL), and the optical density (OD) at 600 nm was adjusted to about 1.0. The lysozyme solution (10 μL) was added to the substrate solution, and the mixture was incubated for 30 min at room temperature. Change in OD per minute was calculated and divided by enzyme concentration to provide a unit number of the enzyme activity. The concentration of lysozyme was determined by measuring the absorbance peak at 595 nm by using Coomassie staining.

## Results

### FTIR Analyses of the FEL-Irradiated Lysozyme Fibrils

**Fig. 2:**
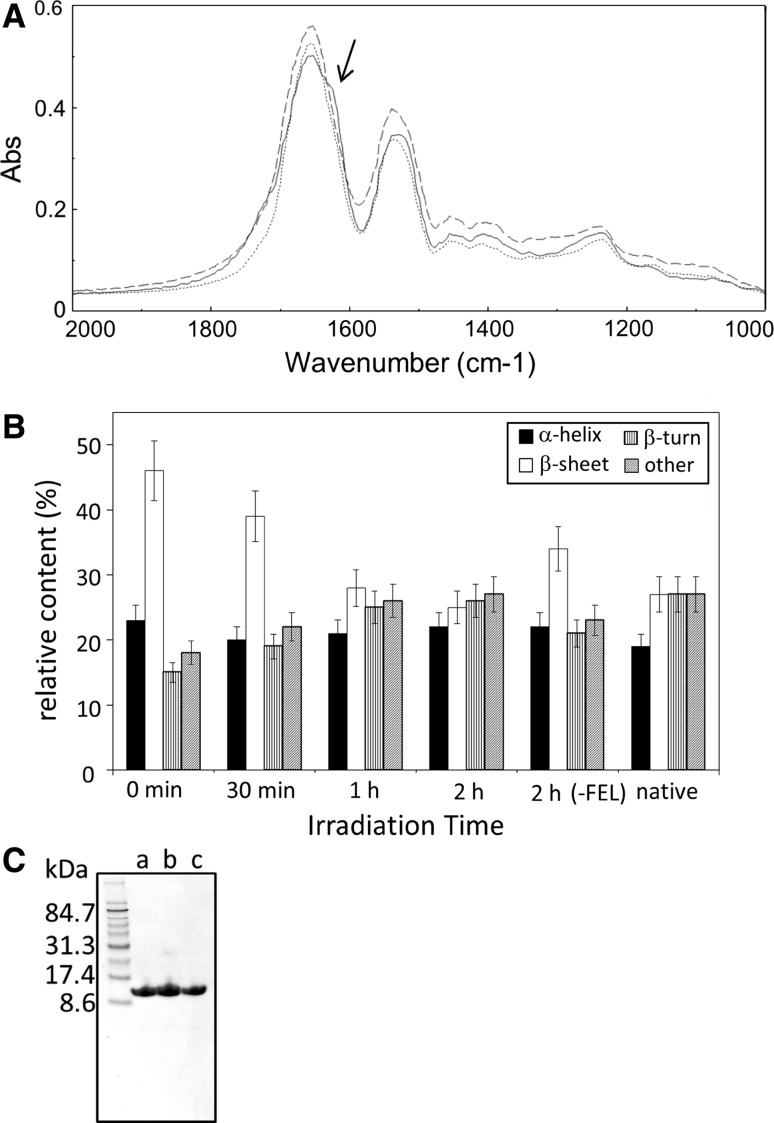
Effect of FEL irradiation on the structural change of lysozyme fibrils. **A** FTIR spectra of lysozyme samples before and after the FEL irradiations at 1,620 cm^−1^ for 2 h. The measurements were operated using KBr-plate and 16 scans. *Solid line* the spectrum of the enzyme before the irradiation; *dotted line* that of the enzyme after the irradiation; *dashed line* that of native enzyme. An *arrow* indicates the wave number targeted by the FEL irradiation. **B** Secondary-structure analyses. The percentages were calculated based on the de-convoluted spectra of amide I bands. “Others” indicate the disordered region. The experiments including FEL irradiation and the following measurement of FTIR spectra were performed five times, and the statistical errors of secondary-structural changes, which were mainly caused by the FEL irradiation conditions, were evaluated to be ±10 % (one standard deviation). **C** SDS-PAGE analyses. The lysozyme samples were loaded on the gel (15 %) without heat denaturing. *a* native lysozyme, *b* fibrils, *c* fibrils following the FEL irradiation

According to the method of Krebs et al. [13], lysozyme fibrils were generated under acidic conditions with high concentration of salt for 20 h at 37 °C, and were precipitated. The FTIR spectrum of the precipitate displayed a shoulder around 1,620 cm^−1^, appearing near the main peak at 1,653 cm^−1^ of amide I band (solid line in Fig. 2A). The β-sheet content in the fibrils was estimated to be in the range of 40–50 %, compared to 25–28 % in the native state, by the analysis of the secondary structure (Fig. 2B, 0 min vs. native). The amide II band was also observed as a broad peak occurring near 1,520 cm^−1^. These spectra of amide I and II regions were determined to be caused by the formation of intermolecular β-sheet structures [2, 9]. Next, the precipitate of fibrils (approximately 0.5 mg in weight) was placed on the glass slide and mixed with 10–20 μL of water at 37 °C, as shown in Fig. 1. Immediately, the mixture was irradiated under atmospheric conditions by the FEL tuned to a frequency of 1,620 cm^−1^ with a pulse energy of 6–8 mJ; one or two drops of water were periodically added to the sample to prevent the fibrils from drying out during the irradiation period. The FTIR spectrum of the irradiated sample indicated that both broad peaks at 1,620 and 1,520 cm^−1^ decreased to nearly the same level as that observed in the native lysozyme after irradiation (Fig. 2A, dotted line). The β-sheet content decreased as the irradiation time increased and achieved almost the same level (25 %) as that of the native one after 2-h irradiation (Fig. 2B). These results indicate that the β-sheet–rich structure refolded into the native structure under the specified irradiation conditions with mid-IR FEL. However, the β-sheet content of the fibrils in the absence of FEL irradiation also decreased during incubation in water [Fig. 2B, 2h (-FEL)]. This observation suggests that spontaneous refolding of fibrils proceeded in the water-suspended mixture during the incubation.

Fragmentation of the peptide backbone of lysozyme did not occur during FEL irradiation, as shown by SDS-PAGE analysis (Fig. 2C). That is, the band of native lysozyme (MW, 14 kDa) was observed between 8.6 and 17.4 kDa of the marker proteins (lane a). In the case of fibrils, one major band was observed at the same position as that of the native protein, and another minor band was observed in small quantities below 31.3 kDa, the putative dimer of lysozyme (lane b). By contrast, following FEL irradiation at 1,620 cm^−1^, one band was seen at the same position as that of the native protein (lane c). The above results indicate that FEL irradiation under the above condition does not destroy the primary structure of lysozyme.

### TEM Analysis

**Fig. 3:**
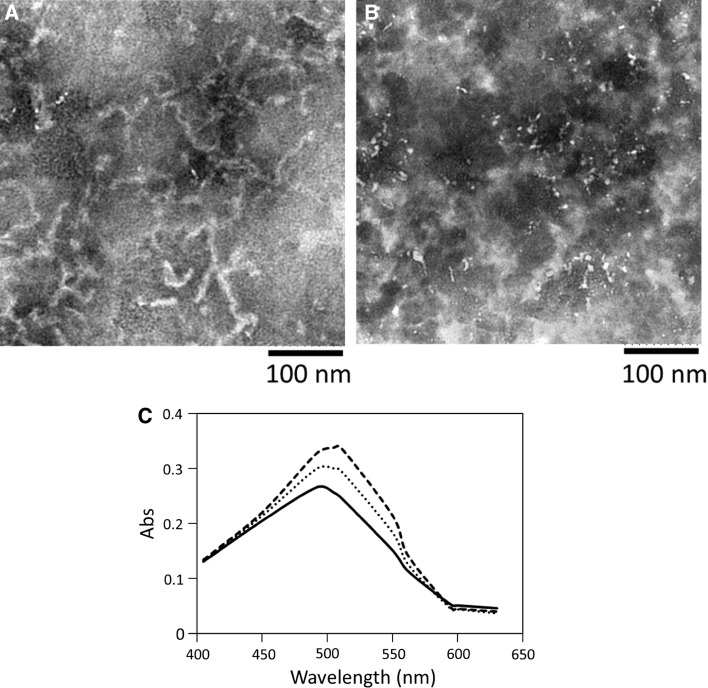
Post-irradiation analyses by TEM and CR assay. **a** TEM image of lysozyme fibrils. The fibrils were formed under the acidic condition and precipitated and dissolved with distilled water. Phosphotungstic acid was used for the negative staining. **b** The image of the fibrils following the FEL irradiation at 1,620 cm^−1^ for 2 h. **c** CR assay. The reagent (0.2 mM) was mixed with the lysozyme solutions (ca. 5.0 mg/mL), and the absorbance values were measured from 400 to 650 nm. *Solid line* the spectrum of CR with native lysozyme; *dashed line* that with lysozyme fibrils; *dotted line* that with the fibrils following the FEL irradiation

Lysozyme fibril formation was also examined by TEM analysis. The fibril material was dissolved in distilled water and mixed with phosphotungstic acid for negative staining. In the TEM image (Fig. 3a), several fibril strings of about 10-nm width but with varying lengths were observed. Following FEL irradiation at 1,620 cm^−1^ for 2 h, the fibril strings decreased in quantity, and small spots with a thickness of several nanometers in diameter were observed, which were predicted to be native globular proteins (Fig. 3b). These results also indicate that the quantity of the fibril structure can be decreased by FEL irradiation.

### CR Assay

Congo Red binding assay was also performed to clarify the effect of FEL on refolding of lysozyme fibrils into the native form (Fig. 3c). The dye is known to bind to amyloid fibrils, and shift the absorbance peak from 490–500 nm to 500–510 nm upon binding [12]. While one peak was observed at 492 nm in the case of native lysozyme bound to the dye (solid line), the peak was shifted to 510 nm when the fibrils were bound to the dye (dashed line). When the dye was mixed with the fibrils following FEL irradiation, the absorbance peak was shifted to near 492 nm, indicating that non-fibrils were more abundant than fibrils (dotted line). Therefore, the number of lysozyme fibrils decreased following FEL irradiation.

### Specificity of the FEL Irradiation on the Refolding of Lysozyme Fibrils

**Fig. 4:**
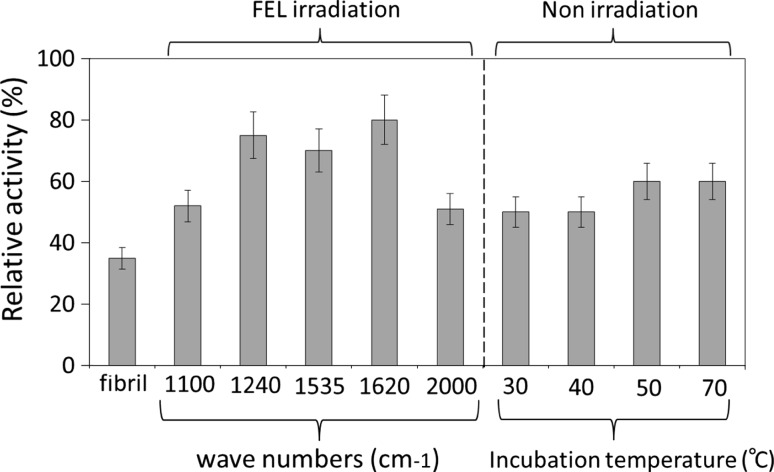
Recovery of enzymatic activities of lysozyme fibrils after the FEL irradiation or heating. The hydrolytic activities of lysozyme samples were measured using the bacterial cell wall extract, and the relative value (%) of each sample against the native activity was shown. The measurements were performed at least three times, and the reproducible average values were described. The wave numbers of FELs were tuned to amide I (1,620), II (1,535), and III (1,240), or non-amide region (1,100 and 2,000), and the irradiations were performed at 37 °C for 1.5 h. For non-irradiation, the fibrils were dissolved in water and heated at 30–70 °C for 1.5 h. Then, the enzymatic activities were measured similarly with the above method. The activity of fibrils before the treatment was shown as a reference

Next, we examined the specificity of FEL on the refolding of fibrils (Fig. 4). The activities of lysozyme fibrils following irradiation by FELs tuned to amide bands (1,620, 1,535, and 1,240 cm^−1^) were compared to those tuned to non-amide regions (1,100 and 2,000 cm^−1^). Further, the effect of FEL on the refolding activity was compared to that of the external heating (30–70 °C) in the absence of FEL. The lysozyme activity was measured using bacterial cell wall extracts, expressed as a relative value (in percent) against the activity of native enzyme. We have evaluated the statistical errors to be about ±10 % (one standard deviation), from three repeated measurements. When the fibrils were irradiated by FEL tuned to amide bands (1,240, 1,535, and 1,620 cm^−1^) with pulse energy of 8–10 mJ, the activities of the generated fibrils were about 70–90 % of the native protein, compared to that of non-treated fibrils, which was only 35 %. By contrast, when the fibril was irradiated at non-amide region (1,100 or 2,000 cm^−1^), the activity was only approximately 60 % at either wave number. These results indicate that lysozyme fibrils could be more refolded by the FEL irradiation tuned to the amide (I, II, and III) bands than that tuned to the non-amide region. The enzymatic activity of the fibrils after thermal incubation at over 50 °C in the absence of FEL irradiation was also found to be about 60 % of the native lysozyme. Although these observations indicate that the fibrils can be refolded by increasing the incubation temperature, also suggest that the FEL-irradiation tuned to amide bands is more effective for the refolding of the fibrils than the thermal incubation at high temperatures.

## Discussion

The FEL system has been used in the field of laser surgery for ablation of pathological tissues [5, 18]. In many instances, protein matrices were destroyed and the higher-order structures of proteins were altered by FEL irradiation. From these studies, it can be inferred that the denatured structure of a protein may also be affected by FEL irradiation. The aggregation processes of proteins into the inactive, denatured forms are, in many instances, associated with the onset of human diseases, such as Alzheimer’s disease. Dissociation of these fibrils into soluble and active forms of the protein is expected to have both mechanistic and clinical applications. In the present study, we applied mid-IR FEL to the process of refolding of amyloid-like fibrils of lysozyme into the native form and observed that FEL irradiation markedly affected the β-sheet content of the protein and promoted the refolding of the fibrils. Although there should be many structural differences between lysozyme fibrils and amyloid β fibrils and it may be difficult to apply the present result using the FEL for reducing the fibrils in vivo, we consider that a possibility indicating that amyloid-like fibrils can be refolded under the FEL-irradiation condition could be found in this study. Oomens et al. [17] suggested that FEL irradiation at the amide I band of the protein affects the ionic interactions between the amide backbone and various ligands. Therefore, in the instance of fibril refolding, we suggest that non-covalent bonds between the β-sheet structures can be affected by FEL irradiation. The vaporization of water with FEL irradiation is also suggested to be a driving force of tissue ablation [28]. In addition, wavenumber-dependent refolding of fibrils could be observed (Fig. 4). It can be estimated that the FEL irradiation at amide absorption heats the fibrils and surrounding water which leads to higher temperatures which drive dissociation of the fibrils and refolding of lysozyme to its active state. Although it can be suspected that the native structure may be also affected by the FEL irradiation, the structural damage of native protein is probably caused by the higher power of FEL more than the present value (max. 15 mJ per macro-pulse) for a long-term irradiation.

In conclusion, refolding of amyloid-like fibrils of lysozyme can be promoted by mid-IR FEL irradiation under mild condition at 37 °C. Although further studies are required to clarify a detailed mechanism of FEL-induced refolding, mid-IR FEL is expected to be useful for dissolving the insoluble amyloid-like fibrils of additional proteins into their active forms.

## Abbreviations

Aβ: Amyloid β
CR: Congo Red
FTIR: Fourier transform infrared spectroscopy
Mid-IR FEL: Mid-infrared free-electron laser
PBS: Phosphate-buffered saline
SDS-PAGE: Sodium dodecyl sulfate polyacrylamide gel electrophoresis
TEM: Transmission-electron microscopy
